# Multisystem failure and death due to extensive hemorrhaging and brain herniation subsequent to a bite by an unidentified snake

**DOI:** 10.1186/s41182-016-0029-2

**Published:** 2016-09-13

**Authors:** N. D. B. Ehelepola, S. M. A. N. Samaranayake, B. M. L. S. Basnayake, C. G. K. Amiyangoda, D. M. U. C. B. Dhanapala, K. L. R. Kalupahana

**Affiliations:** The Teaching (General) Hospital-Kandy, Kandy, Sri Lanka

**Keywords:** Snakebites, Snake envenomation, Sri Lanka, Coagulopathy, Acute kidney injury, Brain hemorrhage, Brain herniation, Russell’s viper, Case report

## Abstract

**Background:**

Snakebites cause considerable morbidity and mortality in tropical and subtropical countries even though existing treatment methods can prevent most deaths if presentation occurs early to hospitals. Envenomation by unidentified snakes is common in central Sri Lanka. Management of such patients is challenging especially if presentation is late.

**Case presentation:**

Here, we report a case of a 52-year-old man from central Sri Lanka who presented late after being bitten by an unidentified snake. He developed `severe coagulopathy, neurotoxicity, acute kidney injury, and rhabdomyolysis. Subsequently, despite of treatment, he died due to extensive hemorrhaging in many organs. A large intracranial hemorrhage lead to fatal brain herniation.

**Conclusions:**

Envenomation by some snake species can severely affect multiple body systems and give rise to fatal brain hemorrhages and brain herniation. Considering the known effects of local snake venom, the responsible species is likely to be Russell’s viper (*Daboia russelii*). We recommend some simple measures to reduce the chances of such deaths in the future.

## Background

Snakebites are a neglected health problem which mainly affects tropical and subtropical countries. At least 100,000 annual deaths occur worldwide due to snakebites [1], and South Asia (which includes Sri Lanka) is the most heavily affected region [2]. Effective treatment exists if patients come early to hospital [1]. In Sri Lanka, the great majority of people have access to a state hospital within a couple of hours and they are provided with treatment free of charge. Bites by unidentified snakes are common in central Sri Lanka [3] and elsewhere. Treating them is more challenging, especially if the patient is presented late to a hospital. Doctors are compelled to guess the probable snake species responsible by comparing patients’ symptoms and physical signs with the known patterns of symptoms and signs caused by bites of snake species in the locality. Information on place and the time of the bite may also give clues about the responsible species. Terrestrial elapids’ and vipers’ venom, respectively, cause predominantly neurotoxicity and coagulopathy. Sea snakes, which also are elapids, have venom causing rhabdomyolysis. However, a combination of these toxicities and toxicities on other systems like the kidneys ensues after envenomation by many snake species.

**Fig. 1:**
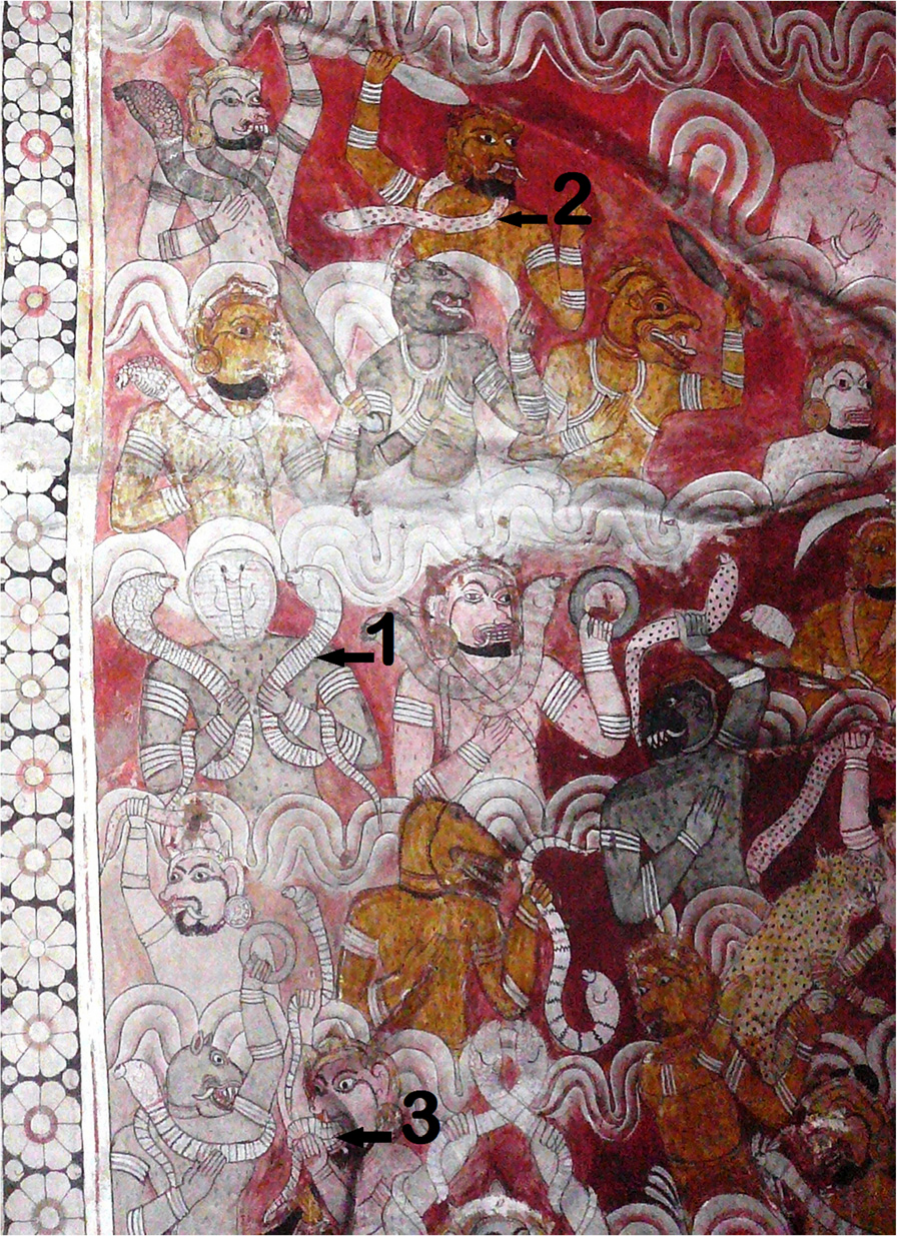
This illustrates a section of the demon troops of Mara who symbolize forces of death (in a spiritual context). Most of them carry venomous snakes as weapons, mostly spectacled cobras which are historically the most feared snake by Sri Lankans.*1* A spectacled cobra-headed demon carrying two cobras. *2* spotted stout body of a Russell’s viper locally known as thith polonga (spotted viper). *3* Slender shorter body and the pattern of bands of the skin indicate kraits. This photo was taken by the first author

Out of the 102 species of snakes known to live in Sri Lanka, approximately half are endemic, 38 species possess venom, and 87 live on land [4]. Only six terrestrial species are known to have venom lethal to humans, and three of those species are vipers. Those vipers are the Russell’s viper (*Daboia russelii*), saw-scaled viper (*Echis carinatus*), and the Merrem’s hump-nosed viper (*Hypnale hypnale*); the last species was categorized as one with lethal venom only recently. The elapids are spectacled cobra (*Naja naja*), common krait (*Bungarus caeruleus*) and Ceylon krait (*Bungarus ceylonicus*). Russell’s viper, spectacled cobra, and common krait contribute to the great majority of hospital deaths [5]. Figure 1 is a photo of a ceiling mural of one of the historic rock caves of the Golden Temple of Dambulla in central Sri Lanka. The mural depicts the Sri Lankan snakes responsible for most snakebite deaths through the ages.

In the experiences of the authors to date and according to published information [3], hump-nosed viper (*H. hypnale* and two other moderately venomous endemic hump-nosed viper species) bites are the most commonly reported in hospital practice in the Central Province.

Data on snakebite victims of Sri Lanka and most other developing countries is only based upon those who venture to hospital. Many cases do not come to hospital so the magnitude of the problem is underrepresented [1, 2, 6]. In Sri Lanka, as in many other countries, many people, especially in rural communities, rely on indigenous medical practitioners for treatment of snakebites and patients who go to them do not get recorded. [1, 6]. However, they come to hospitals if and when symptoms get worse [6]. Fatality rates in hospitals for snake bites have greatly reduced in the recent past in Sri Lanka [5].

## Case presentation

A 52-year-old Sri Lankan man was bitten by an unidentified snake on his left foot whilst he was walking along a village footpath (approximately 900 m above sea level) in the Central Highlands of Sri Lanka at about 21.30 h. He was apparently healthy except for diabetes mellitus which had been diagnosed approximately 1 year ago, for which he took oral hypoglycemics haphazardly and was not on regular follow-up. The snake vanished into the vegetation, he walked home (approximately 250 m), and the family summoned an indigenous doctor similar to how their ancestors had done even though a state hospital was about 4 km away. The indigenous doctor arrived after 30 min, applied a herbal paste over the left foot (the formula, a family secret, has been handed down through the generations), made a drink from karapincha (*Murraya koenigii*) leaves and coconut kernel, gave it to the patient, and chanted some *mantras* (sacred verses). The patient passed loose stools three times.

As he felt a progressive difficulty to open his eyes and had blurred vision approximately 22 h after the bite, he was taken to the nearby hospital. The doctors there noticed bilateral ptosis and gum bleeding, diagnosed systemic envenomation, and transferred the patient to the Teaching Hospital-Kandy by ambulance.

On arrival (24 h since the bite), his left foot was edematous with two fang marks and with a thin topical herbal paste, there was gum bleeding, and the mouth was reddish with paste of betel nuts he had been chewing (Betel nut is an Asian mild stimulant composed of leaves of *Piper beetle*, seeds of *Areca catechu*, and lime). There was bilateral ptosis, external ophthalmoplegia; his pulse rate was 90/min, his blood pressure was 110/80 mm mercury, respiratory rate was 48/min, and a few bilateral crepitations were auscultated in his lungs that disappeared after nebulization with salbutamol (albuterol). Arterial oxygen saturation was 96 % whilst he was on oxygen via a face mask. His abdomen was normal on examination. When an intravenous line was started, there was bleeding from the puncture site. He was conscious, rational, and well-oriented. Both pupils were equal and central and reacted to light. He could lift his head off the bed but could not do so against resistance during the assessment of neck muscle power. In all limbs, his muscle tone, power, and tendon reflexes were normal.

His abnormal laboratory investigation findings are summarized in Table 1.

**Table 1:** The summary of laboratory investigations

Test	Abnormal result/s		Reference range
	Day 1	Day 2	
Whole blood clotting time (WBCT)	>20 min → >20 min	<20 min	<20 min
Total white cell count	21.32 × 10^9^/l (neutrophils 79.8 %)	24.16 × 10^9^/l (neutrophils 88.2 %)	4.0–10.0 × 10^9^/l (neutrophils 50.0–70.0 %)
Platelets	46 × 10^9^/l	138 × 10^9^/l	150–450 × 10^9^/l
Creatine kinase CK2	6792 U/l		<171 U/l
Blood urea	18.6 mmol/l	18.2 mmol/l	2.10–7.10 mmol/l
Serum creatinine	2.4 mg/dl → 3.56 mg/dl	3.4 mg/dl	0.67–1.17 mg/dl
Serum sodium	124 mmol/l → 129 mmol/l	132 mmol/l → 129 mmol/l	132–148 mmol/l
Serum potassium	5.4 mmol/l → 4.9 mmol/l	3.9 mmol/l → 5.2 mmol/l	3.5–5.3 mmol/l
Serum alanine transaminase (ALT)	91 U/l		7–45 U/l
Serum aspartate transaminase (AST)	197 U/l		13–31 U/l
Activated partial thromboplastin time (APTT)	50.5 s	77.2 s	Control 32.0 (30–45) s
International normalized ratio (INR)	3.25	1.70	<1.3
Arterial blood PH	7.2 → 7.32		7.35–7.45
Random blood sugar	356 mg/dl	160 mg/dl	<200 mg/dl

Hemoglobin, hematocrit, serum calcium (albumin corrected), serum magnesium, serum phosphorus values were within normal range, and the chest X ray (taken on admission) was also normal

**Fig. 2:**
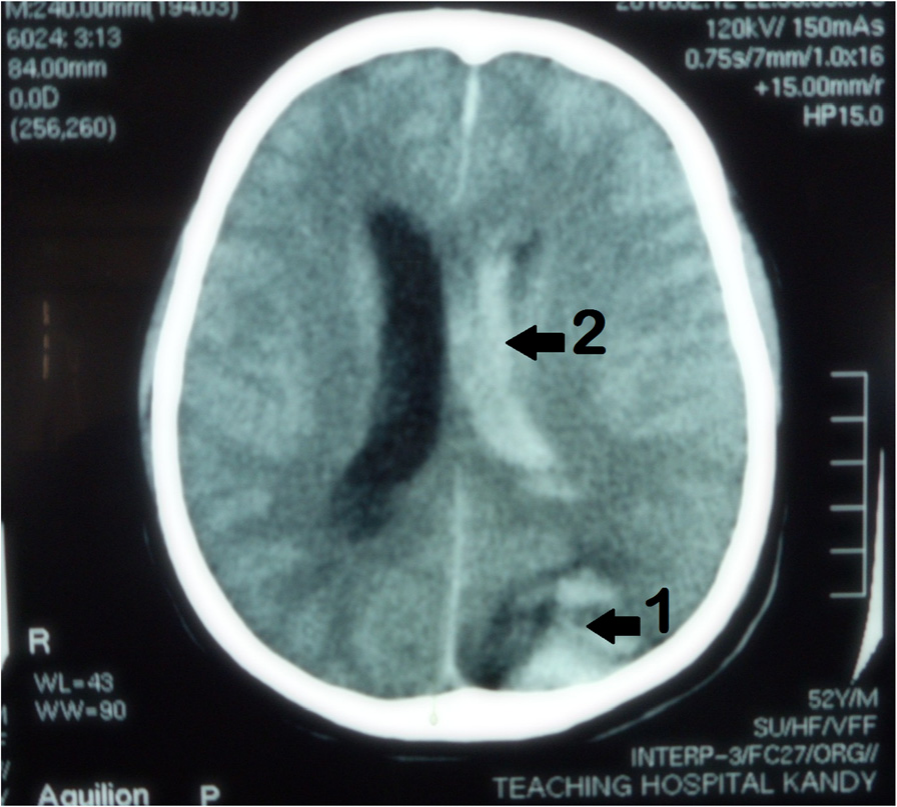
From NCCT-brain scan of the patient, intracerebral hemorrhage in the left occipital region (*arrow1*), to the left lateral ventricle (*arrow2*), and cerebral edema are evident

His blood sugar level was controlled by initial intravenous and subsequent subcutaneous soluble insulin. He once had developed an allergic reaction to pineapples. Ten vials of polyvalent anti-snake venom (ASV) serum (Bharat Serums and Vaccines (ltd), Ambernath, India) were infused under the cover of adrenalin (epinephrine) which improved ptosis and ophthalmoplegia a little. One hundred fifty milliliters of dark urine gradually appeared when his bladder was catheterized, and thereafter, he was anuric. There was muscle tenderness and his serum creatinine kinase level was approximately 40 times the normal upper limit. A test for myoglobinuria was not available. There was no intensive care unit bed available in regional state hospitals. To cover possible secondary bacterial infection from the bite site, intravenous cloxacillin was started. Another 10 vials of ASV were infused as the whole blood clotting time (WBCT) did not improve after initial ASV infusion, and as a result, the patient developed shivering. This was controlled by intravenous chlorpheniramine and hydrocortisone. Later, WBCT improved. As the patient had acute kidney injury, a nephrology referral was done. His platelet count was low so he was hemodialysed for 3 h after being infused with six units of platelets. Twenty-one hours after admission, the patient complained of a headache to the nursing staff and he temporarily responded to 1 gram of oral paracetamol (acetaminophen). Later, he reported he had had a headache at home as well and it subsided in response to paracetamol. Twenty-two hours after hospital admission, bradycardia was detected in the cardiac monitor. An urgent 12-lead electrocardiogram was done as cardio toxicity was also a possibility [7] and only sinus bradycardia was detected. Twenty-five hours after admission (22.30 h), the patient was again reassessed, and considering increasing blood pressure, bradycardia, and recent high activated partial thromboplastin time (APTT) and international normalized ratio (INR) values (although his pupils were equal), an urgent noncontrast CT scan (NCCT) of the brain was carried out. This showed a left occipital intracerebral hemorrhage (ICH) with an intraventricular hemorrhage (Fig. 2), cerebral edema, and a developing right-sided hydrocephalus.

Nevertheless, there were no new external bleedings.

The infusion of mannitol and furosemide was prescribed to control intracranial pressure. We contemplated the infusion of ASV again. A doctor from the neurosurgical team was summoned to see the patient, but immediate surgical intervention was ruled out considering high risk of bleeding and overall condition (American society of anesthesiologists—category 5) of the patient. Before finishing the neurosurgical assessment, the patient developed a cardiac arrest and our attempts to resuscitate him failed.

### Post-mortem findings

**Fig. 3:**
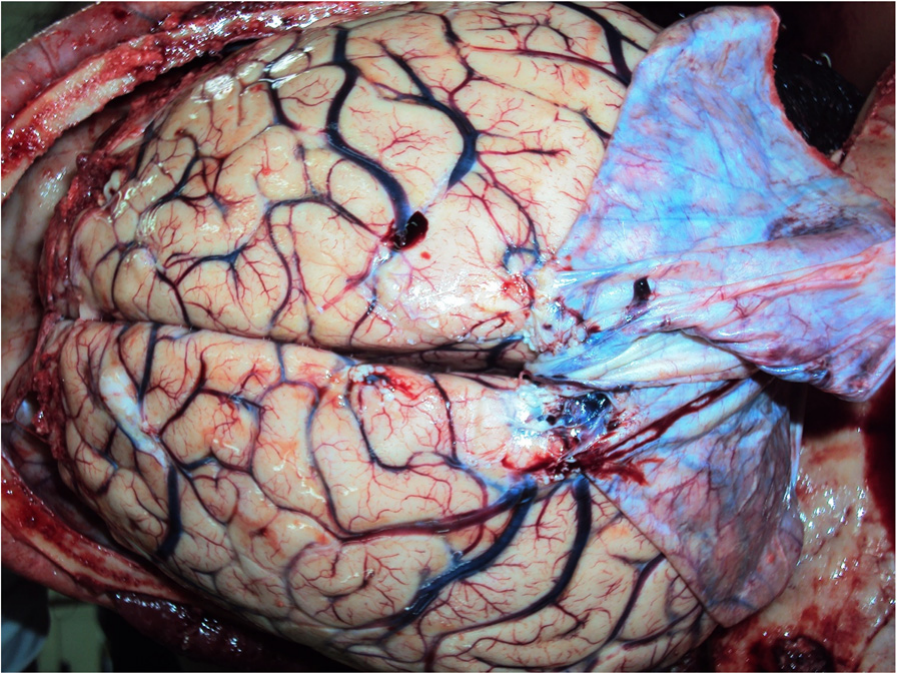
Gross edema of the brain and congested blood vessels can be seen here (cerebral gyri were flattened)

**Fig. 4:**
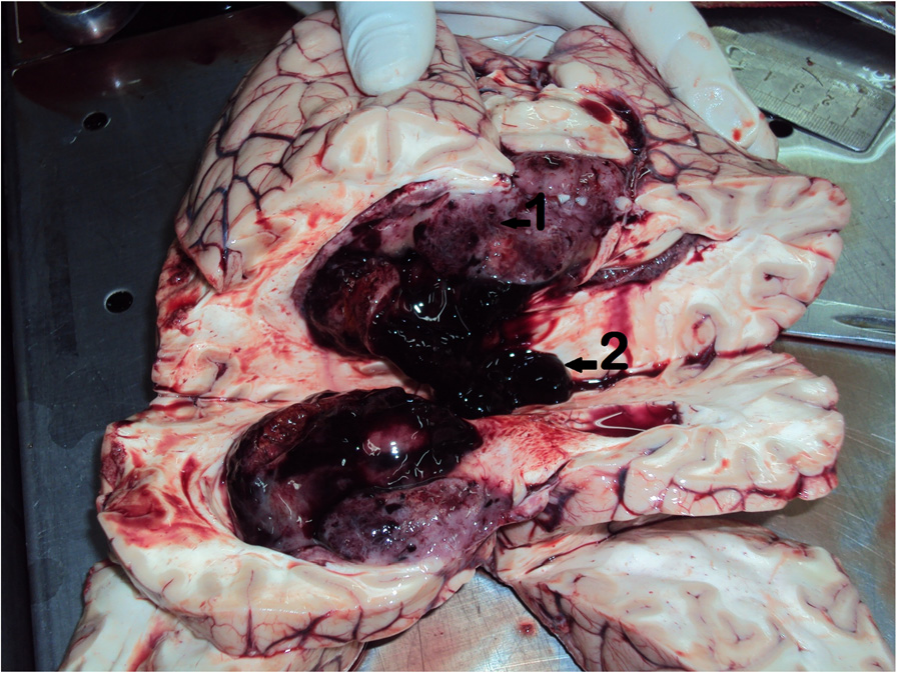
Intracerebral hemorrhages (*arrow1*) that appear to be older than the hemorrhages in the lateral ventricles (*arrow2*) of the brain are seen here

**Fig. 5:**
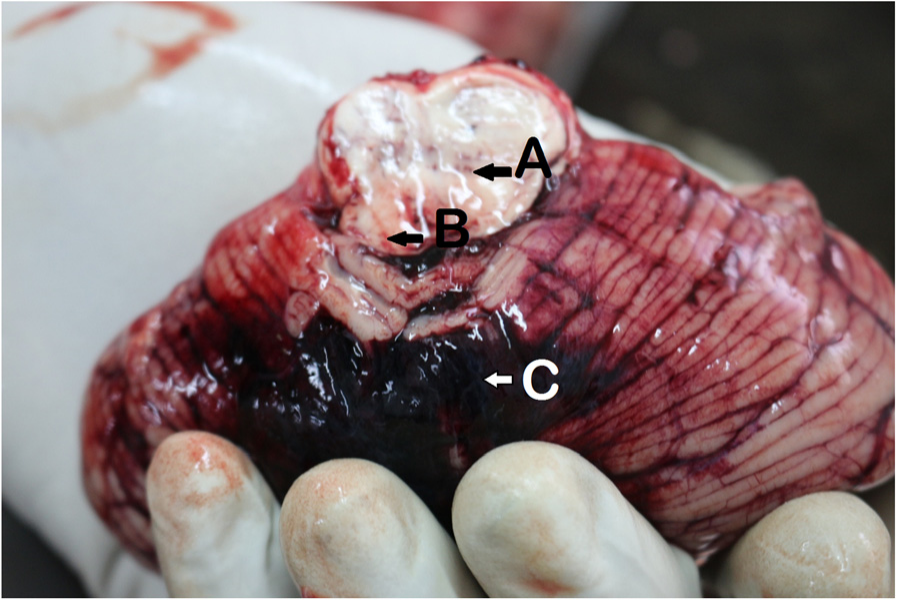
Hemorrhages in the brain stem (*arrow A*), evidence of brain stem herniation (*arrowB*), and basal subarachnoid hemorrhages (*arrowC*) can be seen in this photo

There was gross cerebral edema (Fig. 3) and both supratentorial and cerebellar tonsil herniation of the brain too. There were intracerebral hemorrhages and hemorrhages in the lateral ventricles (Fig. 4) extending to the fourth ventricle. Additionally, brain stem hemorrhages and basal subarachnoid hemorrhages were identified (Fig. 5).

There were hematomas at the bite site and the site where the femoral vein was catheterized for hemodialysis. There were subendocardial hemorrhages in the heart. Both kidneys were edematous. There was blood into both pelvicalyceal systems and hemorrhages in both lungs as well.

### Discussion

We discussed the relevant issues under three subheadings.

#### Identification of the snake species responsible

On admission to our hospital, this patient had features of neurotoxicity, coagulopathy, and local edema at the bite site suggesting systemic envenomation likely to be by a Russell’s viper. Dry bites are rare with Russell’s vipers as they usually deliver a full dose of venom when biting. Saw-scaled viper bites are extremely rare whilst krait bites are rare in the Central Highlands of Sri Lanka [3]. The venom of both deadly local kraits is unlikely to cause coagulopathy and significant local edema. Spectacled cobra venom is unlikely to cause severe coagolopathy or acute kidney failure. Hump-nosed viper venom is not known to cause neurotoxicity despite ischemic strokes being reported post-bite [8]. Subsequent detection of acute kidney injury may be caused by a hump-nosed viper too but more likely to be by Russell’s viper venom. Severe myotoxicity with neurotoxicity is classically associated with sea snakes but the area is inland highlands and sea snake bites do not result marked local reactions. Russell’s viper venom also can cause severe myotoxicity and neurotoxicity [7]. It is uncommon to see all those systems get severely affected as in this patient after a snakebite. The time, venue of incident, and bite to foot also point towards Russell’s viper [3, 7]. However, the patient had no abdominal pain (which is usually associated with serious Russell’s viper envenomation) [3].

**Fig. 6:**
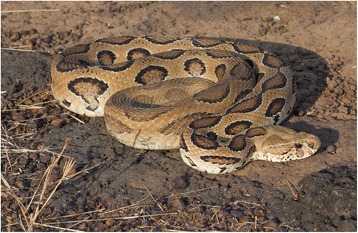
A Russell’s viper (*Daboia russelii*), locally known as “Thith polanga” (meaning spotted viper) and other names. Most Sri Lankans simply call them “polonga” (the viper) since it is the most significant viper they dread

Hemorrhaging in the brain and other organs is known to occur after Russell’s viper bites [2]. Ischemic strokes also were reported after Russell’s viper bites [9]. Russell’s viper venom is a potent agent that activates factor X of coagulation cascades resulting in the consumption of coagulopathy and the activation of the fibrinolytic system [2, 10]. This explains why it can cause both hemorrhages and ischemic strokes. Neurotoxicity due to Sri Lankan Russell’s viper is reported to poorly respond to ASV [7]. However, in this patient, there was mild improvement of ptosis and ophthalmoplegia half an hour after the start of infusion of ASV. In spite of the ASV infusion which occurred twice, he had severe internal bleedings (autopsy). Basal subarachnoid hemorrhage (SAH) that was not in the NCCT brain was detected at autopsy. His headache at home and the finding of older and fresher bleeding to the brain in the autopsy indicate that brain hemorrhages may have started at home and recurred. The basal SAH may have been caused by the spread of intraventricular hemorrhages. We think the possibility of a species other than those six considered is unlikely, and hence, this circumstance is probably a case of Russell’s viper envenomation (Fig. 6).

#### Why he died and possible measures to improve outcome of similar cases

The Indian polyvalent ASV is the only ASV available in Sri Lankan hospitals at present. This covers venoms of the “big four” lethal snakes of the Indian subcontinent namely Russell’s viper, saw-scaled viper, spectacled cobra, and the common krait but not the venom of other species. Although causing frequent reactions (such as the case of this patient), the ASV is fairly effective in preventing death if administered early to counter the venom of those species. The decision to infuse (and repeat) should be made carefully if the snake is unidentified. Late presentation to hospital had reduced the chances of his survival. His walking after the bite may have accelerated systemic spread of injected venom. The availability of an intensive care unit bed and quicker availability of laboratory reports would have made management of this patient much easier. Aside from the hospital admission delay, any beneficial or harmful effects of indigenous doctor’s treatment on his disease process are unknown to us.

This patient was the breadwinner of his family. He and most local victims of snakebites are in productive years of their lives [7], which is another reason for primary prevention of snakebites. He was wearing rubber slippers and walking with the aid of the light of his mobile phone instead of a torch at the time of the bite which made him vulnerable. Education of the public about identification of snakes with lethal venom (using color photos/videos) may also help to reduce incidences like this.

Some clinical manifestations of Sri Lankan Russell’s viper envenomation are reported to be different from that of the same species in continental Asia [7]. A monospecific ASV was developed locally to address this issue but was not very successful [5]. A polyvalent ASV that covers venom of *H. hypnale* (responsible for most snakebites) and at least other five lethal terrestrial snake species of Sri Lanka would have been more useful to treat the patient bitten by an unidentified snake. One ongoing project is developing a polyvalent ASV that covers venoms of local snakes with international help [11].

#### Snake species with venom that can cause similar symptoms

A recent case report from India describes a large intracerebral hemorrhage following a suspected snakebite which was successfully treated with neurosurgery [12]. Brain hemorrhages following envenomation by *D. russelii*, *Pseudonaja textilis*, *Notechis scutatus*, *E. carinatus*, and Bothrops species have been reported from various countries [2, 12, 13]. However, we did not find any reported cases of brain hemorrhages and edema leading to supratentorial and tonsillar herniation of the brain that caused death after being bitten by an unidentified snake. Many snake species with venom with hematotoxicity or myotoxicity and snakes with both toxicities like *D. russelii*, *Oxyuranus scutellatus*, and *N. scutatus* can cause acute kidney injury. There are at least 16 snake species that can cause nephrotoxicity [3, 14]. Snakebites are attributed to up to 70 % of acute kidney injury cases in some Asian countries [14].

## Conclusions

Bites by some snake species rarely cause severe brain hemorrhages and edema that lead to brain herniation and death, and it is useful to keep this possibility in mind when managing patients with severe envenomation. If snakebite victims with coagulopathy complain of headaches, it is worth doing a NCCT scan of the brain, vigorous promotion of using a good torch at night, and wearing footwear that covers the whole foot when walking on rural footpaths especially at night may help to reduce snakebites in high-risk areas. Promotion of hospitalization as soon as possible with the affected limb immobilized may help to reduce morbidity and mortality of snakebite victims. Increasing the number of intensive care beds in state hospitals may help to give better care to patients with serious envenomation.

## Abbreviations

WBCT: Whole blood clotting time
ALT: Alanine transaminase
AST: Aspartate transaminase
APTT: Activated partial thromboplastin time
INR: International normalized ratio
ASV: Anti-snake venom
NCCT: Noncontrast CT scan
ICH: Intracerebral hemorrhage
SAH: Subarachnoid hemorrhage
